# The Mitochondria–Endoplasmic Reticulum Contacts and Their Critical Role in Aging and Age-Associated Diseases

**DOI:** 10.3389/fcell.2019.00172

**Published:** 2019-08-21

**Authors:** Ornella Moltedo, Paolo Remondelli, Giuseppina Amodio

**Affiliations:** ^1^Department of Pharmacy, University of Salerno, Fisciano, Italy; ^2^Department of Medicine, Surgery and Dentistry, “Scuola Medica Salernitana,” University of Salerno, Baronissi, Italy

**Keywords:** aging, age-associated diseases, oxidative stress, senescence, endoplasmic reticulum, mitochondria

## Abstract

The recent discovery of interconnections between the endoplasmic reticulum (ER) membrane and those of almost all the cell compartments is providing novel perspectives for the understanding of the molecular events underlying cellular mechanisms in both physiological and pathological conditions. In particular, growing evidence strongly supports the idea that the molecular interactions occurring between ER and mitochondrial membranes, referred as the mitochondria (MT)–ER contacts (MERCs), may play a crucial role in aging and in the development of age-associated diseases. As emerged in the last decade, MERCs behave as signaling hubs composed by structural components that act as critical players in different age-associated disorders, such as neurodegenerative diseases and motor disorders, cancer, metabolic syndrome, as well as cardiovascular diseases. Age-associated disorders often derive from mitochondrial or ER dysfunction as consequences of oxidative stress, mitochondrial DNA mutations, accumulation of misfolded proteins, and defective organelle turnover. In this review, we discuss the recent advances associating MERCs to aging in the context of ER–MT crosstalk regulating redox signaling, ER-to MT lipid transfer, mitochondrial dynamics, and autophagy.

## Introduction

**FIGURE 1:**
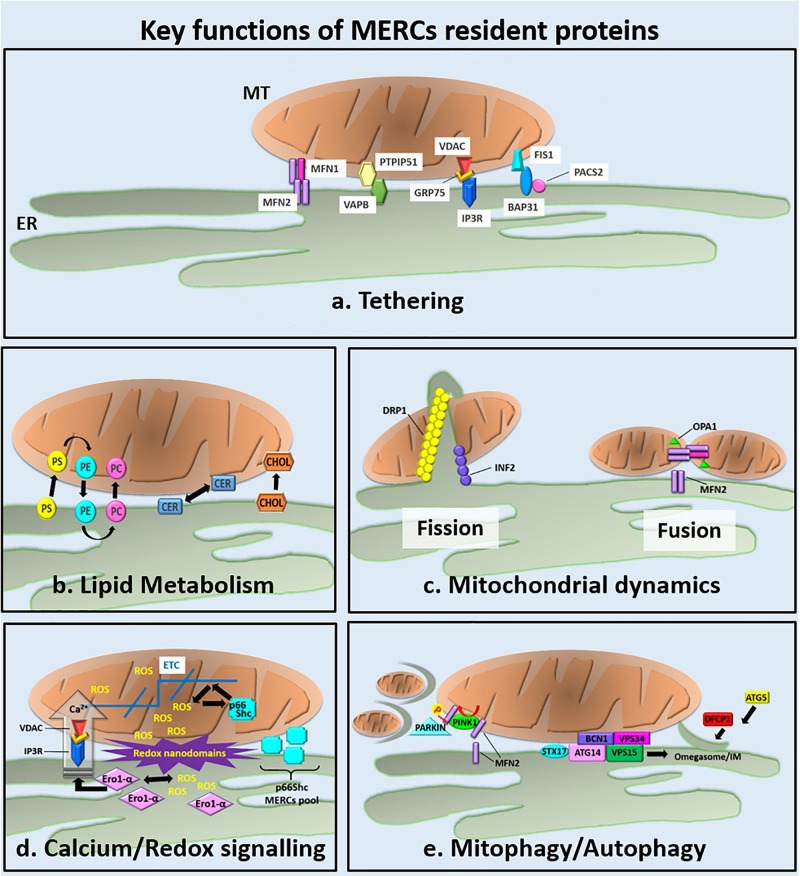
Overview of the key functions of the MERCs resident proteins. **(a)** The main ER–MT tethering factors are described from left to right. Although it is mainly localized at MT, a small amount of Mfn2 is found at ER, where it participates to the tethering of ER to MT through the formation of ER–Mfn2/MT–Mfn2 homodimers or ER–Mfn2/MT–Mfn1 heterodimers (de Brito and Scorrano, 2008; Cosson et al., 2012; Filadi et al., 2015; Naon et al., 2016). VAPB is an integral ER protein and binds the mitochondrial PTPIP51 protein. The reduced or increased expression of IP3R decreases or increases, respectively, the number of ER–MT contacts (Stoica et al., 2014; Gomez-Suaga et al., 2017). The ER-localized IP3R Ca^2+^ channel forms a tether with the mitochondrial Ca^2+^ channel VDAC. Their interaction is mediated by the mitochondrial chaperone 75 kDa Grp75 and modulates Ca^2+^ fluxes from the ER to the mitochondrial intermembrane space (Mendes et al., 2005; Szabadkai et al., 2006). The ER protein BAP31 interacts with the mitochondrial fission protein Fis1. The BAP31–Fis1 complex bridges the ER–MT interface and regulates the mitochondrial induction of apoptosis. The exact function of PACS2 at MERCS is still unknown but its depletion uncouples the ER from MT by inducing the cleavage of BAP31 and MT fragmentation (Simmen et al., 2005; Iwasawa et al., 2011). **(b–e)** Key cellular functions handled at MERCs (see the text for details). **(b)** MERCs are involved in lipid metabolism through the ER–MT exchange of PS, PE, PC, Cers, and Chol (Vance, 2014). **(c)** MERCs appear as sites promoting mitochondrial fission and fusion. During mitochondrial fission, INF2 recruits DRP1 at MERCs mediating the formation of the constriction ring around the mitochondrial outer membrane (Friedman et al., 2011; Chakrabarti et al., 2018). MFN2, the core component of mitochondrial fusion machinery, was found to localize at MERCs, were, together with OPA1, promotes the fusion of mitochondrial membranes. **(d)** ER–MT redox crosstalk occurs at MERCs where different mechanisms are responsible for ROS production: Ca^2+^ flux from the ER to MT through the IP3R/VADC Ca^2+^ channels, the oxidative folding activity of the ER chaperone Ero-1α, and the electron transport promoted by p66Shc at mitochondrial ETC. The high amount of ROS produced at MERCs generates redox nanodomains at ER–MT interface that modulates ER–MT apposition (Debattisti et al., 2017). **(e)** MERCs are emerged as important regulators of mitophagy/autophagy. During mitophagy, the MERCs localized Mfn2 is phosphorylated by PINK1. Phosphorylated Mfn2 recruits parkin that, in turn, mediates MFN2 ubiquitination leading to mitophagy initiation (Bockler and Westermann, 2014). Concomitantly, MERCs have been proposed as sites of autophagy initiation (Hamasaki et al., 2013; Bockler and Westermann, 2014). Upon starvation, the ER-resident protein STX17 recruits ATG14 to the MERCs. ATG14, along with other subunits of the Beclin 1/VPS34 complex, became enriched in the MAM and generates concentrated pools of PI3P necessary for IMs formation and expansion. The omegasome marker DFCP1 was also observed to translocate onto these PI3P-enriched regions with further associated markers like ATG5 (Axe et al., 2008; Hamasaki et al., 2013) contributing to the first steps of autophagosomes assembly.

The existence of MT–ER contacts (MERCs) was demonstrated in the late 50s by electron microscopy (Copeland and Dalton, 1959). Biological functions, for instance modulation of phospholipid transfer and Ca^2+^ interchange were the first functions established for MERCs (Vance, 1990; Rizzuto et al., 1998), but more recently, additional roles, such as the regulation of mitochondrial dynamics (Friedman et al., 2011), inflammasome formation (Zhou et al., 2011), activation of autophagy (Hamasaki et al., 2013), and redox signaling control (Booth et al., 2016) have been charged to these structures. To better define the molecular composition of MERCs different methods, ranging from subcellular fractionations to proteomics and electron microscopy, have been deployed. As a result, a structural and functional equivalence has been established between MERCs and MT-associated membranes (MAMs) (Giacomello and Pellegrini, 2016). The first refers actually to the ultrastructural architecture that can be observed by electron microscopy, whereas the second hints at the molecular composition of fractions derived from ER–MT membranes isolated by subcellular fractionation, respectively. Recently, high-electron microscopy and super-resolution optical microscopy have hugely contributed to the uncovering of the architectural complexity of MERCs (Csordas et al., 2006; Sood et al., 2014; Giacomello and Pellegrini, 2016; Krols et al., 2016; Sezgin, 2017; Chakkarapani et al., 2018). By these methods, MERCs appear as site of parallel juxtaposition between MT and smooth or rough ER tubules at a distance ranging from 10 to 80 nm. In different tissues, the length and the thickness of the contact zones and the protein composition are variable and differently tuned by signaling pathways, which are under the control of apoptosis, ER stress response, or metabolic dysfunction (Rowland and Voeltz, 2012). In addition to that, the increasing number of proteomic studies and the new toolkits utilized have highlighted a novel group of proteins frequently involved in MERCs (Poston et al., 2013; Hung et al., 2017). Some of them retain the function of tethering factors that hold the two organelles in close proximity (Figure 1). The best established ones include mfn2 (de Brito and Scorrano, 2008; Cosson et al., 2012; Naon et al., 2016), the PhosphoAcidic Cluster Sorting protein 2 (PACS2) (Simmen et al., 2005), the complex formed by VAPB, the PTPIP51 (Gomez-Suaga et al., 2017), and the association between the subtype 3 of the 1,4,5-triphosphate receptor (IP3R3) and the mitochondrial VDAC1 (Szabadkai et al., 2006). However, although the growing effort to define the exact protein composition of MERCs, their high plasticity represents such a great challenge for modern biology that their identity in mammalian cells still remains debated (Rowland and Voeltz, 2012).

## Role of MERC*s* in Er-to-Mitochondria Redox Signaling During Aging

Abnormal production of ROS, leading to oxidative damage to DNA, proteins, and lipids, is a key event contributing to aging and age-associated disorders such as neurodegenerative and motor disorders (Krols et al., 2016; Bernard-Marissal et al., 2018; Fasano et al., 2018), cancer (Danese et al., 2017; De Marco et al., 2018; Iorio et al., 2018; Morciano et al., 2018), metabolic syndromes (MetSs) (Theurey and Rieusset, 2017), as well as cardiovascular diseases (CVDs) (Barja, 2014; Holmström and Finkel, 2014; Wang et al., 2018; De la Fuente and Sheu, 2019). According to recent discoveries, almost all intracellular compartments produce ROS, as side effects of metabolic pathways and in a manner depending on the cell type and/or on the pathophysiological state (Brown and Borutaite, 2012; Holmström and Finkel, 2014). When under control, ROS level is important to modulate many physiological events. Instead, an excessive ROS production can affect molecular structures and function of intracellular compartments, ultimately leading to cellular senescence.

Both MT and ER are sites of ROS production, therefore the communication at MERCs participates to the diffusion of the harmful effects of ROS production inside the cell.

The involvement of MT in age-related oxidative stress has been historically assessed by the mitochondrial free radical theory of aging (Barja, 2014). At mitochondrial level, the respiratory chain and, in particular, Complexes I and III components are the main source of superoxide radical anion (${\text{O}}_{2}^{•-}$). Together with the ETC Complexes I and III, other mitochondrial enzymes are known sources of ROS, including cytochrome b5 reductase, monoamine oxidase, α-ketoglutarate dehydrogenase, pyruvate dehydrogenase, and the flavoprotein–ubiquinone oxidoreductase (Balaban et al., 2005). Proteomic studies allowed the quantification of carbonylated proteins that are produced by oxidative stress in relation to aging. According to these analyses, during lifespan, mitochondrial proteins result the over represented ones and also showed the greatest increase in carbonylation (Cabiscol et al., 2014).

More recently, several evidences reveal that also the ER is an important source of ROS even though its impact on intracellular oxidative stress is less prominent compared to the MT (Amodio et al., 2011, 2018; Cao and Kaufman, 2014). Within the ER, ROS is produced predominantly by members of cytochrome P450, NADPH oxidase 4 (Nox4), and by the process of oxidative protein folding mediated by the ER oxidoreductin (Ero1) α and β (Amodio et al., 2018). Little is known about the age-associated modulation of ROS production in the ER. Nevertheless, as revealed by some studies looking at the hepatic tissues of aged mice, there are evidences that ER-resident proteins, such as the molecular chaperones PDI and Bip/Grp78, undergo oxidative damage and progressive dysfunction during senescence (Rabek et al., 2003; Nuss et al., 2008). In any case it is important to point out that the transmembrane protein protein kinase RNA-like ER kinase (PERK), a component of the ER stress/unfolded protein response (UPR) machinery (Cao and Kaufman, 2014; Amodio et al., 2018), is found abundant in MAMs (Verfaillie et al., 2012). Curiously, in this contest, PERK would play the role of tethering factor by making tighter the ER–MT membrane contacts during ER stress to ease Ca^2+^ influx and, as a consequence, the ROS-dependent mitochondrial apoptosis. Thus, this is a further important example, which suggests that MERCs are decisive to mediate inter-organelle signaling leading to decision between cell death or surviving that could take an important part during aging.

Definitely, a large amount of scientific literature demonstrates the involvement of the ER–MT interplay in redox signaling and aging, but the role of MERCs in such a mutual dependency has emerged only in the past years. One example is the redox crosstalk between the ER and MT described for the regulation of Ca^2+^ signaling. In particular, the work by Booth et al. (2016) demonstrated that Ca^2+^ fluxes from ER evoke the production of large amount of ROS from MT cristae that are responsible for the generation of redox nanodomains at ER–MT interface. Such a strongly oxidizing environment, found around MERCs, modulates ER–MT apposition through the mitogen-activated protein kinase (MAPK)-dependent control of mitochondrial mobility (Debattisti et al., 2017) and, ultimately, can affect the function of proteins involved in MERCs.

In our opinion, given the importance of oxidative damage in senescence, the ER–MT redox crosstalk cannot be separated by the age-dependent deterioration of proteins. One example comes from the regulation of ryanodine receptors (RyRs) activity. RyR is the ER Ca^2+^ release channel required for skeletal muscle contraction. Although the localization at MERCs of the RyR has not been defined yet, in the skeletal muscle of aged mice it has been reported how the increased carbonylation and oxidation of RyRs is associated to Ca^2+^ leak, ROS production, and muscle weakness (Andersson et al., 2011). Interestingly, the forced expression of catalase into the MT rescues RyR oxidation and prevents Ca^2+^ leak (Umanskaya et al., 2014). These data suggest the existence of bi-directional communication between the ER-localized RyR and MT that, not surprisingly, occur at MERCs.

Many other proteins known to be part of the MERCs structure are directly involved in the ER/MT redox crosstalk in aging and age-associated diseases.

As an example, a well-established case of MERCs-localized protein involved in redox signaling and aging is the 66-kilodalton (66-KDa) isoform of the growth factor adapter Shc (p66Shc) protein. It is well-known that the 66-KDa Shc isoform, a negative regulator of the epidermal growth factor (EGF)-stimulated MAPK pathway, controls oxidative stress and life span in mammals (Okada et al., 1997; Migliaccio et al., 1999). In addition, p66Shc catalyses the electron transfer from cytochrome c to oxygen in the mitochondrial intermembrane space inducing the formation of H_2_O_2_, which in turn triggers the activation of the mitochondrial dependent apoptotic pathway (Giorgio et al., 2005). The localization of p66Shc at MT can be induced by oxidative stress insults that exert its critical phosphorylation to Ser36 residue and therefore its association to the MT (Migliaccio et al., 1999; Pinton et al., 2007). Thus, one can envisage a feedback loop regulation, where p66Shc is firstly activated by oxidative stress and, in turn, induces mitochondrial oxidative stress and apoptosis. In this context, the capacity to activate apoptosis in response to ROS renders p66Shc a potential life-span determinant. This hypothesis is supported by a large amount of reports. For example, it is known that the enrichment of Ero1-α at MAM fractions is modulated by the redox state of MERCs (Gilady et al., 2010). This event, in turn, can potentiate Ca^2+^ signaling at MERCs through the Ero1-α-dependent production of H_2_O_2_ and the consequent oxidation of the IP3R (Li et al., 2009; Anelli et al., 2012). p66Shc has attracted considerable attention since it was found that p66Shc-knockout mice exhibited extended lifespan, increased resistance to oxidative and hypoxic stress, and a reduced amount of atherosclerotic and ischemic lesions (Trinei et al., 2002; Napoli et al., 2003; Zaccagnini et al., 2004). Additionally, it was found that primary fibroblasts obtained from centenarians, as well as liver, heart, lungs, skin, and diaphragm from adult mice, express higher levels of p66Shc compared to the younger counterparts (Pandolfi et al., 2005; Lebiedzinska et al., 2009). Likewise, work by Pinton et al. (2007) reported that the induced mitochondrial Ca^2+^ uptake inversely correlates with the number of passages of cultured mouse embryonic fibroblasts (MEFs) cells, whereas this was not observed in p66Shc-deficient cells. On the same line, oxidative stress induced mitochondrial fragmentation in wild-type MEFs but not in the p66Shc-deficient cells or in MEFs treated with a blocker of p66Shc phosphorylation at Ser36 (Pinton et al., 2007).

All together, these data suggest that p66Shc is a key player in preserving the mitochondrial fitness and, as a consequence, in regulating cellular physiology and senescence. Nevertheless, it is important to point out that its exact localization within the cell is still undefined. Indeed, p66Shc was previously thought to be a cytosolic protein but, recently, it was found also in different MT compartments, in the MAM fraction and in the plasma membrane-associated membranes (PAMs) (Giorgio et al., 2005; Lebiedzinska et al., 2009). Interestingly, its localization at MAMs is considered as the origin of the mitochondrial p66Shc pool (Lebiedzinska et al., 2009). Moreover, the level of p66Shc in the PAM or in the MAM fractions is a function of the age as reported in animal models, where higher levels of p66Shc are detected in the MAM fraction of older animals, indicating a role for this protein in the “induction” of mitochondrial oxidative stress correlated with aging and senescence (Lebiedzinska et al., 2009).

All together, these data confirm the detrimental outcome of redox signaling in aging and strongly indicate that MERCs take a major part in the accumulation of ROS-induced damages during senescence.

## Lipid Transfer at the Er–Mitochondria Interface as Lifespan Determinant

Historically, lipid transfer from ER to MT was the first function ascribed to MERCs (Vance, 1990). In mammalian cells, the key event in this biological process is the transfer of PS from ER, where it is synthetized by the PS synthase 1 and 2 (PSS1 and PSS2), to the outer surface of the inner mitochondrial membrane. Here, PS-decarboxylase converts PS in PE, which is transferred back to the ER, where it is methylated to produce PC, the most abundant phospholipid in cellular membranes (Flis and Daum, 2013; Vance, 2014). The transfer of PS from ER to MT, which occurs at MERCs, is the rate-limiting step and becomes essential in condition of ethanolamine restriction. Interestingly, new components involved in the direct transfer of phospholipids between ER and MT have been recently described in yeast and defined as ER–MT encounter structures (ERMESs) and mitochondrial contact site and cristae organizing systems (MICOSs) (Aaltonen et al., 2016; Kojima et al., 2016). ERMESs were proposed to facilitate phospholipid transfer between the ER and MT, while MICOSs appeared to be involved in the beginning of close contacts between mitochondrial outer and inner membranes to facilitate mitochondrial PS import and decarboxylation. Remarkably, homologs of ERMESs and MICOSs have been identified also in mammalian cells. However, their role in the lipid transfer at MERCs in mammals is still under definition.

In addition to phospholipids synthesis, MERCs are involved in the metabolism of Chol and Cers (Bionda et al., 2004; Fujimoto et al., 2012). In particular, steroid hormones are produced from Chol in the MT, where the P450 side chain cleavage enzyme (CYP11A1) converts it to pregnenolone, the steroid precursor (Issop et al., 2015). The rate-limiting step in steroid biosynthesis is the availability of Chol, which is synthetized at the ER level and then transferred to MT. As such, the MERCs resident proteins play a pivotal role in dictating and promoting the Chol efflux to MT.

In this context, very attractive is the observation that caveolin-1 (CAV1), a protein involved in Chol intracellular transport and plasma-membrane organization, was found, by mass spectrometry, as a specific component in MAM fractions. At this level, CAV1 controls Chol levels (Bosch et al., 2011; Sala-Vila et al., 2016). Therefore, taking also in consideration the massive presence of Chol at the ER–MT interface, CAV1 plays a fundamental role. Indeed, the amount of Chol present in MAMs is particularly elevated compared to the ER and MT content, so that lipid rafts-like microdomains are present in MAM (Hayashi and Fujimoto, 2010). Moreover, the level of Chol at ER–MT interface seems to be critical for the integrity and function of MERCs. In this regard, it was found that aberrant Chol accumulation at these ER subdomains, due to CAV1 genetic deficiency, leads to reduced MERCs physical extension (Bosch et al., 2011; Sala-Vila et al., 2016). In agreement with previous data, these observations show that Chol depletion strengthen ER and MT association and reduce *de novo* synthesis of PS in association to the increase of PE (Fujimoto et al., 2012). Additionally, MERCs also accommodate enzymes needed for synthesizing Cer (Bionda et al., 2004; Stiban et al., 2008). Since increased mitochondrial Cer levels are associated with the permeabilization of the outer mitochondrial membrane and the initiation of apoptosis (Stiban et al., 2008), MERCs represent again crucial modulators of cellular lifespan (Stiban et al., 2008).

Mitochondria–endoplasmic reticulum contacts coordinate lipid membrane composition that, in turn, is a determining factor for cellular lifespan and aging as assessed since when the “membrane theory of aging” was postulated (Zs-Nagy, 1997). In few words, according to this theory aging is directly correlated to the membrane level of unsaturated fatty acids that are more sensitive to peroxidative damage (Pamplona et al., 2002). Indeed, accumulating evidences corroborate this theory and show that decreasing lipid unsaturation contribute positively to lifespan (Puca et al., 2008).

Another effective strategy to control aging and age-related diseases is the caloric restriction leading to a reduced peroxidation index of membrane fatty acids (Lambert et al., 2004; Lopez-Lluch and Navas, 2016).

The best-established example of association between fatty acids unsaturation and lifespan regulation is provided by cardiolipin (CL). CL is predominantly present in the inner mitochondrial membrane, where it governs crucial mitochondrial functions, including the activity and organization of respiratory chain, the regulation of mitochondrial dynamics and apoptosis, through the retention of cytochrome c (Claypool and Koehler, 2012; Hsu and Shi, 2017). CL is a dimeric phospholipid consisting of four acyl chains characterized by mono- or di-unsaturated chains bearing 16–18 carbons, which predispose CL to be highly susceptible to oxidative damage (Schlame et al., 2005; Hsu and Shi, 2017). Indeed, depletion of CL and remodeling of its fatty acids have been associated to aging. In particular, a significant increase in more unsaturated CL fatty acids, predominantly arachidonic and docosahexaenoic acid, was found in the heart of aged, compared to younger mice (Lee et al., 2006). Interestingly, one of the CL remodeling enzymes is the MAM-enriched enzyme acyl-Coa:lysocardiolipin acyltransferase 1 (ALCAT1), which catalyses the “bad” remodeling of CL, since ALCAT1 incorporates CoA loaded with long-chain highly unsaturated fatty acyl chains (Cao et al., 2004). This ALCAT1-mediated pathological remodeling has a broad impact of mitochondrial function, autophagy, and MAM structure, and has been implicated in aging and age-related diseases (Lee et al., 2006; Petrosillo et al., 2008; He and Han, 2014; Hsu and Shi, 2017). Accordingly, ALCAT1-knockout mice have reduced susceptibility to the onset of age-related diseases including obesity, diabetes, hepatosteatosis, and brain dysfunction (Hsu and Shi, 2017). Another MAM-enriched enzyme, the stearoyl-CoA desaturase 1 (SCD1) is involved in the regulation of membrane saturated/mono-unsaturated fatty acids levels and seems to contribute to aging. In particular, its ER-colocalizing partner diacylglycerol *O*-acyltransferase (DGAT2) was found overexpressed in the skin of aged individuals (Mitchell and Thompson, 1990; Man et al., 2006). In addition, inhibition of SCD1 was associated to reduced accumulation, composition, and saturation of cellular membrane phospholipids, leading to impaired autophagy and autophagosome formation (Ogasawara et al., 2014; Janikiewicz et al., 2015).

Moreover, as detailed later on in this review, MERCs can contribute with an additional mechanism to lifespan determination, which is the regulation of lipid composition (essential) for the autophagy initiation.

## Mitochondrial Dynamics at MERC*s* in Aging

Biological aging is a multifactorial process defined as the time-dependent breakdown in the ability to efficiently regenerate tissues and organs. Such a progressive transformation drives to hypofunctional capacity to counteract cell stress (known as homeostenosis) consequent to the exposure to environmental or endogenous agents. Genomic instability, telomere shortening, epigenetic alterations, impairment of proteostasis, altered nutrient sensing, mitochondrial dysfunction, cellular senescence, stem cells depletion, and altered cell-to-cell communication have been proposed as molecular hallmarks of aging (Lopez-Otin et al., 2013). All these events are under the influence of genetic, epigenetic, and environmental factors thus explaining different course of age-related decline of individuals having the same chronological age. Hence, mitochondrial dysfunction is among the acknowledged hallmarks of aging (Lopez-Otin et al., 2013) and the modulation of mitochondrial fission and fusion dynamics is crucial mechanisms engaged by the cell to cope with the decline in mitochondrial activity associated to mitochondrial DNA damage, accumulation of misfolded protein aggregates, or exposure to stress (Sun et al., 2016). Recently, a wide area of research highlights the participation of MERCs to the regulation of MT dynamics and biogenesis (Friedman et al., 2011; Westermann, 2011; Korobova et al., 2013), pointing up to an additional role of MERCs in the biogenesis of aging.

Mitochondria form a highly dynamic network with morphology varying from fragmented to filamentous as the result of the combination of fusion and fission events (Westermann, 2010). Under physiological conditions, the balance between fusion and fission is necessary to optimize mitochondrial function and quality control. However, when the mitochondrial bioenergetic state became critical, fusion or fission events must be modulated in order to isolate damaged MT or to maximize mitochondrial function and, therefore, to prevent cell degeneration and senescence (Twig et al., 2008; Westermann, 2010). Interestingly, proteins involved in mitochondrial fission and fusion were found significantly enriched in MAM fractions (Friedman et al., 2011; Schon and Area-Gomez, 2013).

Indeed, the Drp1, a mitochondrial adaptor involved in the recruitment of the key fission proteins, was found at MERCs (Arasaki et al., 2015; Elgass et al., 2015). At the molecular level, Drp1 is the crucial protein promoting mitochondrial fission, together with its two adaptor proteins, namely the mitochondrial fission factor (Mff) and the mitochondrial Fis1 (Frank et al., 2001; Westermann, 2010). Interestingly, MERCs participate directly in the early steps of mitochondrial fission, by wrapping around the MT and initiating the mitochondrial constriction required for fission and facilitating the recruitment of Drp1 on the mitochondrial outer membrane (Westermann, 2010). In addition, later studies have showed that an ER-bound protein, the INF2 is required for the recruitment of Drp1 (Korobova et al., 2013; Chakrabarti et al., 2018; Steffen and Koehler, 2018). This event requires two steps: the polymerization of actin at ER–MT microdomains and the amplification of Ca^2+^ efflux from the ER to MT. Both mechanisms are crucial to mediate both the formation of the constriction ring around the mitochondrial outer membrane and to increase the ER–MT contact area, which, in turn, is essential to ensure the execution of mitochondrial fission.

Besides, these observations highlight the existence of a positive feedback between MERCs and MT during fission and this crosstalk is essential in critical situations that require the elimination of MT by mitophagy, as discussed in the next section.

On the other hand, MERCs are equally important for mitochondrial membrane fusion. The core components of the mitochondrial fusion machinery are Mfn1 and Mfn2, along with the OPA1. MFNs are GTPase embedded on the outer mitochondrial membrane and required for the tethering and subsequent fusion of the two lipid bilayers constituting the outer mitochondrial membranes. Following to the realization of the outer membrane fusion, OPA1 mediates the fusion of the inner mitochondrial membranes (Chan, 2006; Westermann, 2010). Interestingly, Mfn2 was found to localize not only at the outer mitochondrial membrane but also at the ER membrane and at MERCs (Koshiba et al., 2004).

From the molecular standpoint, the localization of Mfn2 at MERCs generates multiple effects. As mentioned above, the MERCs-localized Mfn2 seems to be required for tethering MT to the ER and to stabilize MERCs structure through the formation of tight Mfn2–Mfn1 multimer (de Brito and Scorrano, 2008). In support to this notion, the ablation or silencing of Mfn2 expression in mammalian cells disrupts ER morphology and loosens ER–MT interactions and, as a consequence, mitochondrial Ca^2+^ uptake (de Brito and Scorrano, 2008; Naon et al., 2016). However, there are contrasting reports showing that ablation of Mfn2 increases ER-to-MT Ca^2+^ transport (Cosson et al., 2012; Filadi et al., 2015). These discrepancies are probably due to the multiple roles played by Mfn2 at MERCs in different cell types and circumstances and/or to the different methodologies deployed to analyze ER–MT juxtaposition (Filadi et al., 2018).

## Regulatory Role of MERC*s* in Mitophagy

Autophagy exerts a protective role against cellular senescence through the elimination of damaged organelles and intracellular protein aggregates (Rubinsztein et al., 2011; Ranieri et al., 2018). Interestingly, MERCs have been proposed recently as platforms for autophagy initiation and function (Hamasaki et al., 2013; Bockler and Westermann, 2014) on the basis of their role in modulating lipid composition of ER–MT interface. In particular, the artificial increase of PE was found to regulate positively the autophagic flux and, thus, to extend significantly the lifespan in yeast, mammalian cells, and flies (Rockenfeller et al., 2015). In addition, MAM lipid-rafts microdomains and the GD3 ganglioside were reported to participate in the initial organelle scrambling activity that finally leads to the formation of autophagosome (Garofalo et al., 2016).

Besides its role as tethering factor, Mfn2 has emerged as an important regulator of mitophagy, the selective degradation of MT during autophagy activation (Chen and Dorn, 2013; Bockler and Westermann, 2014). In particular, during mitophagy PINK1-phosphorylated Mfn2 functions as a receptor for parkin that, in turn, mediates MFN2 ubiquitination, as a signal to mark damaged MT recruitment and ubiquitination leading to mitophagy initiation. Thus, giving that Mfn2 localizes at MERCs, it is conceivable to speculate that MERCs is primarily involved and participate to mitophagy, rather than to fission and fusion processes.

Studies performed in yeast have provided an interesting model for the role of MERCs in the removal of aged MT from the cell. Indeed, during yeast cells mitosis, tethering activity of MERCs is essential to segregate maternal MT and accumulate toxic protein aggregates, separating those from the MT acquired by the bud, which are largely free of aggregates (Mogk and Bukau, 2014; Zhou et al., 2014). Interestingly, this mechanism, which account for a strategy to rejuvenate cellular environment, involves MERCs and is gradually lost by cells with advanced replicative age, suggesting the participation of MERCs in the mitochondrial quality control (Zhou et al., 2014). More interestingly, in human mammary epithelial cells, a similar event was observed following cellular division. In mammary cells, fine-tuned fission events allow daughter cells, which must maintain stemness properties, to receive newly synthetized MT, while the daughter cells, undergoing to differentiation, receive aged MT (Katajisto et al., 2015). As a consequence, this mechanism is settled to preserve the regenerative capacity of the tissue and prevent senescence. Similarly, recent works have revealed how MERCs were spatially linked to the mitochondrial DNA synthesis both in human and yeast (Murley et al., 2013; Lewis et al., 2016). In other words, it was found that MERCs coordinate replication of mitochondrial nucleoids with mitochondrial fission in order to distribute the proper nucleoids into the daughter MT. Altogether, these evidences confirm the involvement of MERCs in the modulation of mitochondrial fission as a strategy to cope with the establishment of cellular aging.

Giving the crucial role of Mfn2 in ER–MT tethering, mitochondrial fusion, and mitophagy, it is not surprising the increase of experimental evidences and studies linking Mfn2 to aging and age-related diseases. Notably, all the processes related to mitochondrial dynamics ascribed to Mfn2 are important to optimize mitochondrial function and avoid senescence and degeneration. Indeed, Mfn2-knockout in MEFs, cardiomyocyte, and neurons, impairs mitophagy leading to damaged MT accumulation, cell death, and tissue degeneration. Remarkably, these pathological mechanisms involving Mfn2 dysfunction are found in numerous age-related diseases, such as Alzheimer, Parkinson, diabetes, and cardiomyopathies (Filadi et al., 2018).

Not by coincidence, a progressive reduction of Mfn2 that caused impaired mitophagy and accumulation of dysfunctional MT has been reported in the skeletal muscles of aging mice, linked to sarcopenia (Sebastian et al., 2016). In the PolG mice model of premature aging, obtained by expressing a proofreading-deficient version of mtDNA polymerase gamma (PolG mice), mouse cells displayed higher level of the mitochondrial fission protein Fis1, in parallel with increased mitophagy, which likely contributes to the sarcopenic phenotype observed in premature aging (Joseph et al., 2013). In contrast, wild-type aged mice were characterized by higher level of Mfn2 and Mfn1 and reduced levels of Fis1, suggesting increased mitochondrial fusion and reduced mitophagy, probably in response to the physiological accumulation of mitochondrial DNA mutations in the aged muscles. Similarly, increased mitochondrial fusion was also found in senescent mesenchymal stromal stem cells that showed higher levels of Mfn1 and OPA1, together with increased mitochondrial mass and ROS, compared to younger cells at lower passages (Stab et al., 2016).

To summarize, stimulating autophagy in animal models can certainly ameliorate several aging-associated phenotypes. Collectively, these data indicate that the beneficial effects derived from lifespan extension regimens can (at least in part) be explained by the induction of mitophagy. Future studies should provide further insights into how these mechanisms intersect with the mitophagy pathway in order to maintain mitochondrial fitness *in vivo*.

## Function and Dysfunction of MERC*s* in Aging-Related Human Diseases

Aging favors the development of different kind of disorders such as neurodegenerative (Krols et al., 2016; Bernard-Marissal et al., 2018), MetS (Theurey and Rieusset, 2017), as well as CVDs (Barja, 2014; Holmström and Finkel, 2014; Wang et al., 2018; De la Fuente and Sheu, 2019) and cancer (Danese et al., 2017; Morciano et al., 2018).

Among neurodegenerative diseases, late onset Alzheimer and Parkinson’s diseases are the most recurrent age-related disorders. Deranged ER–MT interplay is a common hallmark of neurodegenerative disorders and several studies demonstrate that such pathologies correlate with structural and functional alterations of MERCs (Area-Gomez et al., 2009; Zampese et al., 2011; Cali et al., 2012, 2013). More interestingly, many proteins associated to neurodegenerative disorders are found in the MAMs fractions, although the significance of their presence in MERCs is still under investigation (Area-Gomez et al., 2009; Guardia-Laguarta et al., 2014).

Being the site of β-amyloid peptide (Aβ) production (Schreiner et al., 2015), MERCs play important role in AD pathogenesis. The release of Aβ occurs at MERCs throughout the processing of the amyloid precursor protein (APP) by the γ-secretase complex, composed by the Presenilin 1 and Presenilin 2. In genetic types of AD, mutated Presenilin 2 proteins affect ER–MT connections and their related functions (Zampese et al., 2011). Similar perturbations have been observed also in APP transgenic mouse models as well as in neuronal cells treated with Abβ (Hedskog et al., 2013). In truth, the presence AD-related proteins at MERCs produces quite self-contradicting effect. As a matter of fact, in some cases mutant AD proteins generate a significant up-regulation of MERCs functionality and number (Cali et al., 2012; Ottolini et al., 2013). Intriguingly, even the e4 allele of apolipoprotein E (ApoE4), the most common risk factor for AD-related senile dementia (Liu et al., 2013), has been shown to upregulate the activity of MERCs (Tambini et al., 2016). Instead, in other cases AD proteins generate a decrease of ER–MT tethering (Hedskog et al., 2013).

Analogously, it has been shown that PD-related proteins, such as α-synuclein, Parkin, and protein deglycase (DJ-1), promote ER–MT connections (Cali et al., 2012, 2013; Ottolini et al., 2013; Guardia-Laguarta et al., 2014). In particular, α-synuclein is among the protein found in MAM fractions, where it binds very stably to lipid rafts domain of mitochondrial membranes (Guardia-Laguarta et al., 2014). Likewise, the effect of a-synuclein mutations on the MERCs structure is contradictory. In fact, at least one report showed that expression of mutant forms of α-synuclein decreased ER–MT contacts, whereas another one the result was an increased number of MERCs in PD cells (Cali et al., 2012).

In synthesis, the variable response of MERCs to either AD- or PD-related proteins suggests the requirement of more work to better define the molecular events underlying MERCs function in the different pathological circumstances.

Many evidences show that ER stress is a common hallmark in neuronal diseases such as AD and PD, but also in Huntington’s disease (HD) and amyotrophic lateral sclerosis (ALS) (Remondelli and Renna, 2017). Different conditions can induce the accumulation of unfolded proteins within the ER lumen leading to ER stress (Amodio et al., 2011; Wang and Kaufman, 2016). This state initiates an adaptive response, known as the UPR composed by three integrated pathways: the PERK, the inositol requiring enzyme 1 (IRE1), and the activating transcription factor 6 (ATF6) pathways of the UPR (Walter and Ron, 2011). UPR pathways get started to re-establish proteostasis or, in the case of an unsuccessful recovery, to induce cell death. Several evidences demonstrate a tight link between the ER stress response and neurodegenerative disorders and indicate the UPR pathways as true therapeutic target for such diseases (Remondelli and Renna, 2017).

Remarkably, many reports show that MERCs are closely linked to ER stress and UPR. Indeed, this was shown for MERCs tethering factors such as MFN-2 and for the VAPB (Kanekura et al., 2006; de Brito and Scorrano, 2008; Gkogkas et al., 2008; De Vos et al., 2012; Muñoz et al., 2013; Stoica et al., 2014). In addition, MERCs accommodate essential ER chaperones such as GRP78/BiP, calnexin, calreticulin, ERp44, ERp57, and the Sigma 1 receptor (Mori et al., 2013). Interestingly, along with ER chaperones, MERCs also host the UPR transducers IRE1 and PERK. In particular at MERCs, IRE1 interacts with Sig1R to mediate ER-to-MT survival (Hayashi and Su, 2007; Mori et al., 2013). Furthermore, at MERCs, PERK acts either as tethering factor or as a regulator of ROS transport and, as a consequence, transmission of the apoptotic signaling (Verfaillie et al., 2012). Remarkably, its activity is negatively controlled by the MERCs tethering factor Mfn2 (Muñoz et al., 2013).

In recent years, many compounds have been identified for their ability to reduce or induce ER stress but their effect on MERCs has not been yet examined (Remondelli and Renna, 2017). As we previously discussed, in many of neurodegenerative diseases, the PERK signaling pathway is overactive and this event is considered responsible for neuronal cell death in either AD or PD models. As a consequence, suppression of PERK signaling by using specific PERK inhibitors (i.e., GSK2606414) has neuroprotective effect (Moreno et al., 2013). Interestingly, in AD, PERK activation is correlated to enhanced memory loss and β-amyloidogenesis, which occurs at the ER–MT interface. Remarkably, *in vivo* models of AD, memory impairment is restored by silencing PERK expression (Ma et al., 2013). Similarly, genetic or chemical inhibition of IRE1 signaling retains a protective role against AD by reducing the expression of APP and, as a consequence, βA deposition (Duran-Aniotz et al., 2017).

More interestingly, PERK inhibition was also shown to reduce the MFN contacts at the ER–MT interface. Recently, in an early-onset PD model (PARK20) (Fasano et al., 2018), in which PERK is constitutively activated inhibition of PERK phosphorylation by GSK2606414 generates beneficial effects on the ER stress mitochondrial dysfunction (Amodio et al., 2019). Intriguingly, in pink1 and parkin mutants PD models, suppression of PERK signaling produces neuroprotective effect, most probably by reducing the MFN contacts with the ER that cause enhanced ER stress signaling (Celardo et al., 2016).

All together, these evidences convincingly reinforce the concept that strategies planned to reduce ER stress levels may impact positively on neurodegenerative diseases and strongly stimulate further investigation to identify in MERCs components the most advantageous molecular targets for therapeutic intervention.

Another age-related condition is the MetS, a clinical state affecting about 20% of the aged population in technologically advanced countries. MetS that is characterized by the concomitant presence of cardiometabolic risk factors such as obesity, insulin resistance, dyslipidemia, and hypertension (O’Neill and O’Driscoll, 2015). Several studies underlined the effect of such risk factors on the ER–MT membrane connections. Also in this regard, MERCs organization and function shows different response. As an example, in animal models of obesity, liver cells show increased number of MERCs which in turn causes mitochondrial Ca^2+^ overload followed by mitochondrial dysfunction (Arruda et al., 2014). Instead, in hypothalamic cells the reduction of MERCs obtained by the ablation tethering factor Mfn2 results in ER stress-induced leptin resistance, hyperphagia, reduced energy expenditure, and finally obesity (Schneeberger et al., 2013).

Insulin play essential role in aging and structural integrity of MERCs is a crucial requirement for efficient insulin pathway (Tubbs et al., 2014) as revealed by the observation that depletion of MERCs structural components impairs insulin signaling. Alternatively, overexpression of MERCs proteins increases insulin signaling, as well as pharmacologic rescue of insulin sensitivity re-establishes ER–MT connections, indicating the reciprocal control of both processes (Tubbs et al., 2014). In addition, and as we would expect, ER stress contributes to impaired insulin synthesis in pancreatic β cells and insulin resistance (Shang et al., 2014), suggesting that the pharmacological inhibition of the ER stress pathway could ameliorate insulin deficiency.

Cardiovascular diseases are often associated to MetS and, in particular to obesity, insulin resistance and type 2 diabetes (T2D). In cardiomyocytes, MERCs control of mitochondrial Ca^2+^ uptake is essential to guarantee insulin signaling. As a result, altered cardiomyocyte metabolism and insulin resistance are associated with cardiac hypertrophy (Kolwicz and Tian, 2011; Gutiérrez et al., 2014). Interestingly, in either patients or in animal models of pulmonary artery hypertrophy (PAH), deficiency of MERCs tethering factor Mfn-2 disrupts ER–MT membrane contacts and contributes to pulmonary artery smooth muscle cells hyperproliferation (Ryan et al., 2013).

Essential hypertension is one of the most frequent disturb in the aging. One possibility by which hypertension and other CVDs might arise is the endothelial dysfunction (ED) associated with aging through enhanced oxidative stress. Several studies describe the interconnection between ER stress, UPR, and oxidative stress (OS) in the pathogenesis of ED-derived CVDs (Amodio et al., 2018). Thus, in ED, ER stress and the UPR pathways represent a promising system to test new molecules and develop new therapeutic methodologies for the treatment of ED in aged patients.

Finally, the progressive deterioration of physiological organ function occurring during aging is a primary risk factor for cancer development. Cancer cells exhibit altered expression of proteins, including oncogenes that directly affect MERCs functionality and, in particular, the ER–MT Ca^2+^ transport (Marchi et al., 2014; Stewart et al., 2015). This perturbation results in various characteristics of cancer cells such as resistance to apoptosis, deregulation of cell proliferation, metastatic activity, and a metabolic rewiring. Generally speaking, this happens throughout the expression of oncogenes, such as Mcl-1, Bcl-2, and Bcl-XL, which, by diverse mechanisms, prevent mitochondrial Ca^2+^ overload and cancer cell death (Bittremieux et al., 2016). This event happens by enhancing the IP3R-dependent Ca^2+^ outflow and eluding the ER-dependent mitochondrial Ca^2+^ overload upon stress conditions. On the other hand, expression of tumor suppressor genes, encoding for example the protein phosphatase and tensin homolog (PTEN), PML, BRCA2, favors Ca^2+^ transfer from the ER to MT thus having as consequence pro-apoptotic effects on cancer cells (Bononi et al., 2013; Marchi et al., 2014).

Thus, MERCs are advantageous sites where chemotherapy, hormone therapies, targeted cancer drugs, and bisphosphonates or other anti-tumoral therapies (De Marco et al., 2018; Iorio et al., 2018) might operate to interfere with the function of oncogenes or to restore ER–mitochondrial Ca^2+^ transfer in order to re-establish apoptosis sensitivity of cancer cells or inhibit pro-tumorigenic effects.

As a final point, MERCs participate to the regulation of many functions perturbed in the various age-related diseases. Further examinations of MERCs alterations in these disorders could certainly help to discover novel therapeutic targets to restore the correct ER–MT interplay and prevent the defects by which the diverse pathological features might develop.

## Concluding Remarks and Perspectives

Despite the fact that all the findings we argued are on their own significant, the identification of further mechanisms involved in the control of MERCs function in health and disease will supply critical information on how such a conserved function is regulated and will hopefully provide us with an even more detailed picture of the molecular environment at MERCs. This could, ultimately, pave the way for the discovery of novel therapeutic targets. Indeed, the identification of pharmacological therapies for age-associates dysfunctions remains an ambitious task in biomedical science and such a necessity will become even more serious in consideration of the increased lifespan estimated in the future for the human population.

Overall, the findings discussed in this review confirm that aging is indeed associated to mitochondrial dysfunction as a consequence of the dysregulation of mitochondrial fission, fusion, and mitophagy. Notably, MERCs, holding many of the proteins involved in these processes, may participate directly to the development or prevention of MT-mediated deterioration of cellular physiology, which is observed during aging.

In this context, the identification of novel components and additional factors involved in the MERCs dependent control of cross-talks between ER and MT could be employed to find more pharmacological approaches to be utilized to attenuate or delay the onset of age-associated diseases.

Certainly, a better understanding of the molecular events leading to pathological state for each specific age-related condition is critical in order to adopt the targeting of MERCs-dependent pathways for the cure of age-related disorders. Therefore, additional work should be addressed to identify putative MERCs targets in order to develop novel drugs for the treatment of specific clinical conditions. Finally, identifying cell type-specific regulators of the MERCs could be another approach to precisely modulate the mitochondrial fitness in the desired cellular context (i.e., tissue, organ), without affecting the homeostasis of other cells. For instance, the identification of the MERCs components selectively operating in neuronal cells would be helpful to discover more specific and potentially safe targets in the context of neurodegenerative diseases.

## Author Contributions

All authors drafted the manuscript. OM generated the illustration. GA and PR wrote the manuscript.

## Conflict of Interest Statement

The authors declare that the research was conducted in the absence of any commercial or financial relationships that could be construed as a potential conflict of interest.

## Abbreviations

ATG 14/5: autophagy related 14/5
BAP31: B-cell receptor-associated protein 31
BECLIN-1: BCL-2-interacting protein
Ca^2+^: calcium
Cer: ceramide
Chol: cholesterol
DFCP1: double FYVE-containing protein
Drp1: dynamin-related protein
ER: endoplasmic reticulum
Ero1- α: ER oxidoreductase 1 alpha
ETC: electron transport chain
Fis1: fission 1 protein
Grp75: glucose-regulated protein 75
IM: isolation membranes
INF2: inverted formin 2
IP3R: inositol 1,4,5-trisphosphate receptor
Mfn1/2: mitofusin-1/-2
MT: mitochondria
OPA1: optic atrophy protein 1
p66Shc: 66 kDa proto-oncogene Src homologous-collagen homolog
PACS2: phosphofurin acidic cluster sorting protein 2
PC: phosphatidylcholine
PE: phosphatidylethanolamine
PI3P: phosphatidylinositol 3-phosphate
PINK1: PTEN-induced putative kinase 1
PS: phosphatidylserine
PTPIP51: protein tyrosine phosphatase interacting protein-51
ROS: reactive oxygen species
Stx17: syntaxin 17
VAPB: vesicle-associated membrane protein-associated protein B
VDAC: voltage-dependent anion-selective channel
VPS34/15: vacuolar protein sorting-associated protein 34/15.
